# Phosphorylated EGFR expression may predict outcome of EGFR-TKIs therapy for the advanced NSCLC patients with wild-type EGFR

**DOI:** 10.1186/1756-9966-31-65

**Published:** 2012-08-18

**Authors:** Fen Wang, Shuhang Wang, Zhijie Wang, Jianchun Duan, Tongtong An, Jun Zhao, Hua Bai, Jie Wang

**Affiliations:** 1 Department of Thoracic Medical Oncology, Peking University School of Oncology, Beijing Cancer Hospital & Institute, Beijing 100036, China

**Keywords:** EGFR phosphorylation, EGFR mutation, Non-small cell lung cancer

## Abstract

**Background:**

EGFR mutation is a strong predictive factor of EGFR-TKIs therapy. However, at least 10% of patients with EGFR wild-type are responsive to TKIs, suggesting that other determinants of outcome besides EGFR mutation might exist. We hypothesized that activation of phosphorylated EGFR could be a potential predictive biomarker to EGFR-TKIs treatment among patients in wild-type EGFR.

**Method:**

Total of 205 stage IIIb and IV NSCLC patients, tissue samples of whom were available for molecular analysis, were enrolled in this study. The phosphorylation of EGFR at tyrosine 1068 (pTyr1068) and 1173 (pTyr1173) were assessed by immunohistochemistry, and EGFR mutations were detected by denaturing high performance liquid chromatograph (DHPLC).

**Results:**

Among 205 patients assessable for EGFR mutation and phosphorylation analysis, 92 (44.9%) were EGFR mutant and 165 patients (57.6%) had pTyr1173 expression. Superior progression-free survival (PFS) was seen after EGFR-TKIs therapy in patients with pTyr1068 expression compared to pTyr1068 negative ones (median PFS 7.0 months vs. 1.2 months, *P* < 0.001). Inversely, patients with pTyr1173 had a shorter PFS (4.8 months VS. 7.7 months, *P* = 0.016). In subgroup of patients with wild-type EGFR, pTyr1068 expression positive ones had a significantly prolonged PFS (4.2 months vs.1.2 months *P* < 0.001) compared with those without pTyr1068 expression. Sixteen patients with both wild-type EGFR and pTyr1068 who responded to EGFR-TKIs had median PFS of 15.6 months (95%CI: 7.28-23.9).

**Conclusion:**

pTyr1068 may be a predictive biomarker for screening the population for clinical response to EGFR-TKIs treatment; especially for patients with wild-type EGFR.

## Background

The epidermal growth factor receptor (EGFR) is frequently over-expressed in non-small-cell lung cancer (NSCLC) (32–81%) and is taken as a promising target for NSCLC treatment [1,2]. The representative drugs, such as Gefitinib and Erlotinib, exhibit superior clinical efficacy compared to best supportive care or standard chemotherapy [3,4]. Prior studies have indicated presence of EGFR mutation is a robust predictor of increasing sensitivity to tyrosine kinase inhibitors (TKIs) and is associated with improved progression-free survival with TKIs [5-9]. Interestingly, about 10%-20% of advanced NSCLC patients with wild-type EGFR also benefit from EGFR-TKIs [10-12]. This raises the question whether there are some other predictors beyond EGFR mutation that can reliably identify patients with wild-type EGFR who could benefit from TKIs therapy.

EGFR is a 170 kDa tyrosine kinase receptor consisting of an extracellular ligand-binding domain, a transmembrane lipophilic domain, and an intracellular tyrosine kinase domain and the C-terminus region with multiple tyrosine residues [13]. Ligand binding to EGFR results in homo- or hetero-dimerization, activation of the highly conserved intracellular kinase domain and autophosphorylation of tyrosine residues by γ-phosphate from ATP. The phosphorylated Tyr serve as docking sites of a range of proteins, whose recruitment activate downstream signaling pathways including Ras/Raf/mitogen-activated protein kinase (MAPK) pathway, extracellular signal-regulated kinase (ERK), phosphatidylinositol 3-kinase (PI3K)/Akt pathway, signal transduction and activator of transcription (STAT), and other pathways. ERK1 and ERK2 regulate cell growth and proliferation, whereas Akt and STAT specifically regulate cell survival and apoptosis [14-19]. Five autophosphorylation sites in the EGFR have been identified, all of which are clustered at extreme carboxyl-terminal 194 amino acids. Among these sites, tyrosine (Tyr) 1068, Tyr1148, and Tyr1173 are major sites, whereas Tyr992 and Tyr1086 are minor sites [20]. Distinct downstream signaling cascades are initiated by EGFR depending on its phosphorylation pattern. Phosphorylation at Tyr1068, can bind GAB-1 or Grb2, and subsequently activate their downstream signaling pathways [18,21]. Phosphorylation of Tyr1173 leads to interaction with Shc and phospholipase Cγ (PLCγ), which are involved in activation of MAPK signaling pathway [22].

Numerous preclinical studies have revealed that somatic mutations of the EGFR gene constitutively enhanced EGFR tyrosine kinase activity and receptor autophosphorylation [23-25]. This suggests that regulation of receptor's tyrosine phosphorylation is critical for modulation of the cellular effects of activated EGFR. Recent data shows both mutation and activation status, defined by phosphorylation, might have a strong impact on clinical course [26-28]. One of the predominant C-terminal phosphorylation sites of EGFR is Tyr1068, which used to represent ligand-induced activation of EGFR. Another site, Tyr1173, provides conflicting and confusing information of its correlation with EGFR mutations and predictive value to TKIs therapy [29-31].

Based on the fact that at least 10% of patients with EGFR wild-type respond to TKIs, it is critical to identify potential biomarkers which are helpful to select this subgroup of patients for EGFR-TKIs therapy. In this study, we hypothesized that activation of phosphorylated EGFR could provide predictive information to clinicians and serve as supplement to EGFR mutations for screening patients eligible for TKIs therapy, especially those without EGFR mutations.

## Patients and method

### Patients

205 patients with locally advanced and advanced NSCLC(stage IIIb and IV) treated in Beijing Cancer Hospital from January 2005 to June 2010 were enrolled. All patients had tumor tissues available for biomarkers analysis. Nineteen patients got samples from surgical resection, and others from biopsy. 194 patients received EGFR-TKIs as monotherapy (including 148 in gefitinib therapy and 57 in erlotinib therapy), and had complete clinicopathologic documents. Treatment of Gefitinib (250 mg) or Erlotinib (150 mg) alone daily continued until disease progression, unacceptable toxicity, or patients’ refusal. All patients provided written informed consent and a separate consent for optional provision of tumor samples for biomarker analysis. The study protocol was approved by the Institutional Ethic Committee at Beijing Cancer Hospital.

### Study design

The study was designed to explore potential value of EGFR phosphorylation in predicting clinical response to EGFR-TKIs treatment. Tumor specimens were obtained at initial diagnosis. Clinical data were sealed during laboratory analysis until all data were evaluated. Recorded variables included age, sex, smoking history, pathology, eastern cooperative oncology group (ECOG) performance status, stage at diagnosis, treatments, and toxicities. Efficacy evaluation included best response, objective response rate (ORR), disease control rate (DCR), progression-free survival (PFS) and overall survival (OS).

### Assessments

Tumor assessments were performed at baseline and every eight weeks until investigators documented disease progression or unacceptable toxicity. Clinical responses to TKIs including complete response (CR), partial response (PR), stable disease (SD) and disease progression (PD) were evaluated according to Response Evaluation Criteria in Solid Tumors (RECIST) [32]. PFS was defined as time from beginning of TKIs treatment to PD or death, and OS was defined as time from beginning of TKIs to death. An independent radiologist (Dr. N.W.) assessed all films, who was blind to EGFR biomarker status.

### Tissue samples

Tumor samples from primary sites were routinely fixed in 10% formalin and embedded in paraffin. Serial sections were used for EGFR mutation analysis and phosphorylated EGFR immunohistochemistry.

### DNA extraction and EGFR mutation detection

**Figure 1:**
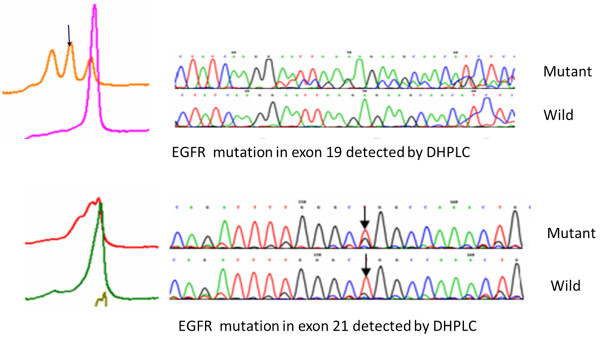
EGFR mutation detected by DHPLC.

Paraffin-embedded biopsy tissues were source of genomic DNA using E.Z.N.A FFPE DNA Kits (OMEGA, USA). EGFR mutation analyses were performed by DHPLC (Figure 1) according to the method described by our colleagues, Bai et al. [33].

### Immunohistochemistry detection

Phosphorylated EGFR protein expression status was assessed by immunohistochemistry using primary antibodies purchased from Cell Signaling Technology (Danvers, MA); Phospho-EGFRTyr1068 (Cad no. 2236) and Phosphors-EGFRTyr1173 53A5 (Cad no. 4407). Immunohistochemical staining was performed according to the manufactures instructions. A commercially available positive control, Signal Slide Phospho-EGF receptor IHC Control (Cad no. 8102) from Cell Signaling was used to validate each anti-phosphoprotein antibody.

**Figure 2:**
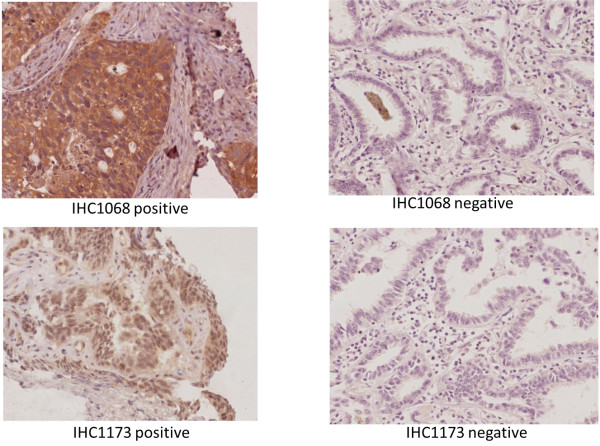
Phosphorylation of EGFR at tyrosine 1068 (pTyr1068) and 1173 (pTyr1173).

Two pathologists independently quantified staining. Every tumor was given a score according to the intensity of cytoplasmic staining (no staining = 0, weak staining = 1, moderate staining = 2, strong staining = 3) and percent of stained cells (0% = 0, 1–10% = 1, 11–50% = 2, >50% = 3). (Figure 2).

Scoring was performed three times per case for three distinct fields, and then three scores were averaged. The average scores for intensity and population were summed, and summed scores above three were categorized as positive in this study.

### Statistical analysis

All statistical procedures were performed with SPSS statistical software, version 16.0 (SPSS Inc., Chicago, IL, USA). The categorical variables were compared using the Pearson’s *X*2 test or the Fisher’s exact test where appropriate. Multivariate analysis was performed using a logistic regression model. The time to event variables (i.e., duration of OS and PFS) and the median OS and PFS were calculated using Kaplan-Meier estimation. Comparisons between different groups were made using the log-rank tests. Multivariate analysis was carried out using the stepwise Cox regression model. Two-sided P values of less than .05 were considered statistically significant. The 95% CIs for odds ratios and frequencies were calculated as exact CIs.

## Results

### Patient characteristics

Among 205 eligible patients, 99 males and 74 patients were active or former smokers. Median age was 61, range from 28 to 84. Adenocarcinoma (ADC) was the predominant histology (169/205) and most of patients were stage IV (168/205). All patients had tissue sample assessable for EGFR mutation analysis and pTyr1068 detection, whereas 156 samples were assessable for pTyr1173 detection. All patients received EGFR-TKIs therapy, including 54 patients as first-line therapy (22 patients harboring EGFR mutation and 32 patients having no indication of chemotherapy due to poor PS or/and severe systematical disease), the median follow-up time for the patients was 12.4 months (range 0.4 to 77.2 months). Baseline patient demographics and disease characteristics are listed in Table 1.

**Table 1:** Baseline demographic characteristics and clinical outcomes for each biomaker

**Parameter (no.of patients, %)**		**EGFR mutation**			**pTyr1068**			**pTyr1173**		
		**+**	**-**	***p***	**+**	**-**	***p***	**+**	**-**	***p***
Total case		92(44.9)	113(55.1)	-	164(80.0)	41(20.0)	-	95(57.6)	70(42.4)	-
Age	<75	85(45.9)	100(54.1)	0.479	148(80.0)	37(20.0)	0.598	86(58.5)	61(41.5)	0.615
	≥75	7(35.0)	13(65.0)		16(80.0)	4(20.0)		9(50.0)	9(50.0)	
Gender	Male	40(40.4)	59(59.6)	0.135	77(77.8)	22(22.2)	0.276	48(57.8)	35(42.2)	0.536
	Female	52(49.1)	54(50.9)		87(82.1)	19(17.9)		47(57.3)	35(42.7)	
Somking history	Never*	59(50.9)	57(49.1)	0.024	94(81.0)	22(19.0)	0.394	49(51.6)	46(48.4)	0.102
	Ever	26(35.1)	48(64.9)		58(78.4)	16(21.6)		38(63.3)	22(36.7)	
	Unknown	7(46.7)	8(53.3)		12(80)	3(20)		8(80)	2(20)	
Histology	ADC	76(45.0)	93(55.0)	0.541	134(79.3)	35(20.7)	0.414	75(55.1)	61(44.9)	0.152
	Non-ADC	16(45.7)	19(54.3)		29(82.9)	6(17.1)		19(67.9)	9(32.1)	
	Unknown	0	1(100)		1(100)	0		1(100)	0	
Disease stage	III	20(57.1)	15(42.9)	0.078	26(74.3)	9(25.7)	0.249	18(60)	12(40)	0.841
	IV	71(42.3)	97(57.7)		136(81.0)	32(19.0)		77(57.5)	57(42.5)	
	Unknown	1(50)	1(50)		2(100)	0		0	2(100)	
TKIs theraphy	Total	89(45.9)	105(54.1)	-	154(79.4)	40(20.6)	-	90(57.7)	66(42.3)	-
Line	First	22(27.4)	32(30.5)	0.623	42(27.3)	12(3.00)	0.843	48(57.8)	35(42.2)	0.365
	Second	67(72.6)	73(69.5)		112(72.7)	28(70.0)		47(57.3)	35(42.7)	
Best response	CR	0(0)	0(0)	<0.001	0(0)	0(0)	<0.001	0(0)	0(0)	0.028
	PR	43(50.0)	17(17.0)		58(39.5)	2(5.1)		25(27.8)	25(37.9)	
	SD	29(33.7)	42(42.0)		57(38.8)	14(35.9)		33(36.7)	30(45.5)	
	PD	14(15.7)	41(38.3)		32(20.8)	23(56.1)		32(35.6)	11(16.7)	
ORR	CR + PR	43(50.0)	17(17.0)	<0.001	58(39.5)	2(5.1)	<0.001	25(27.8)	25(37.9)	0.123
DCR	CR + PR + SD	72(83.7)	59(59.0)	<0.001	115(78.2)	16(41.0)	<0.001	48(57.8)	35(42.2)	0.007
	PD	14(16.3)	41(41)		112(72.7)	28(70.0)		47(57.3)	35(42.7)	
PFS (months)	Median	8.8	2.1	0.024	7	1.2	<0.001	4.8	7.2	0.016
	95%CI	6.11-11.42	0.89-3.24		5.14-8.86	0.96-1.51		2.37-7.23	3.69-11.85	

Abbreviations: EGFR, epidermal growth factor receptor; pTyr, phophorylated tyrosine; CR, complete remission; PR, partial response; SD, stable disease; PD, progressive disease; ORR, objective response rate; DCR, disease control rate; PFS, progression-free survival; *Never-smoker refers to patients had never smoked in their lifetime.

### Biomarker associated clinical outcomes

#### EGFR mutation

In total cohort of 205 patients assessable for EGFR mutation detection, 92 (44%) were positive for EGFR mutation, including 53 in exon 19, 35 in exon21 and 4 double mutations. The univariate analysis indicated never-smoking status was significantly related (*P* = 0.002) to the presence of EGFR mutation (Table 2).

**Table 2:** Correlation between pTyr1068 expression and clinical outcomes stratified by EGFR mutational status

**Parameter (no.of patients, %)**		**EGFR mutation**					
		**Positive**			**Negative**		
pTyr1068		+	-	p	+	-	*p*
Total		84	8	-	80	33	-
TKI therapy		78	8	-	69	31	-
ORR(CR + PR)		53.8(42/78)	12.5(1/8)	0.029	23.2(16/69)	3.2(1/31)	0.01
DCR	CR + PR + SD	85.9(67/78)	62.5(5/8)	0.118	69.6(48/69)	35.5(11/31)	0.001
	PD	14.1(11/78)	37.5(3/8)		30.4(21/69)	64.5(20/31)	
PFS(months)	Median	9.1	4.6	0.224	3.6	1.2	<0.001
	95% CI	6.25-11.94	0.00-11.53		1.03-6.30	1.00-1.46	

Abbreviations: EGFR, epidermal growth factor receptor; pTyr, phophorylated tyrosine; CR, complete remission; PR, partial response; SD, stable disease; PD, progressive disease; ORR, objective response rate; DCR, disease control rate; PFS, progression-free survival.

Of 194 patients who received EGFR-TKIs therapy, 54 (27%) patients received EGFR-TKIs as first-line therapy and 140 (73%) patients as second- or more-line. 60 patients (31%) experienced PR, 71(37%) patients got SD and 63(32%) had PD. No CR was observed. The ORR and DCR of EGFR-TKIs treatment were both higher in patients with EGFR mutations than those without EGFR mutation; ORR was 50.0% (43/89) vs. 17.0% (17/105) *P* < 0.001, DCR was 83.7% (72/89) vs. 59.0% (59/105) *P* < 0.001. In a multivariate analysis involving tumor histology, smoking status, sex, and tumor stage, EGFR mutation was an independent factor for tumor response (OR 0.18, 95% CI 0.09 to 0.38, *P* < 0.001) (Table 1).

**Figure 3:**
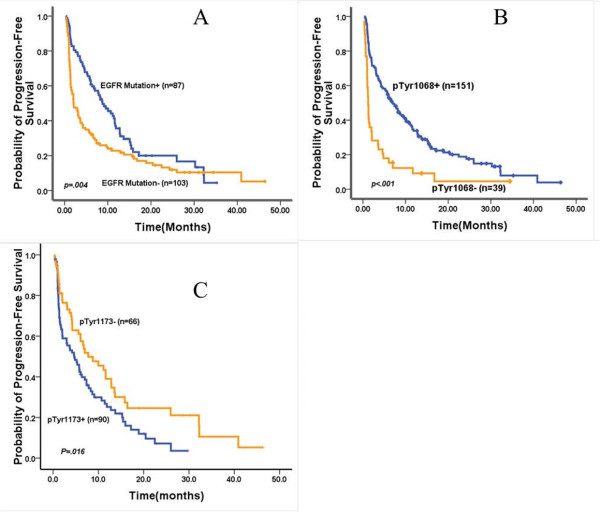
Progression-free survival curves according to epidermal growth factor receptor mutational status (A), phosphorylated tyrosine (pTyr) 1068 expression (B), pTyr1173 expression (C).

PFS was significantly different between patients with EGFR mutation and those without EGFR mutation (Figure 3). Patients with mutation had a median PFS of 8.8 months v 2.1 months for patients without EGFR mutation (*P* = 0.024). Evaluation of OS was available for no more than 50% deaths (85/194) at the last follow-up.

#### pTyr1068 expression

Of 205 assessable patients, 164 (80.0%) had EGFR phosphorylated at Tyr1068. The proportion of patients with pTyr1068 expression was similar across different demographic characteristics (Table 1). Among 194 patients receiving EGFR TKIs, there was a significant difference in ORR or DCR between pTyr1068 expression positive and negative patients; ORR 39.5% (58/154) vs. 5.1% (2/40) *P* < 0.001, DCR 78.2% (115/154) vs. 41.0% (16/40) *P* < 0.001(Table 1). Patients with pTyr1068 expression had a prolonged PFS of TKIs treatment compared with those with unphosphorylated Tyr1068 (7.0 months vs. 1.2 months, *P* < 0.001, Figure 3). A logistic model further confirmed the significant correlation between pTyr1068 and response (OR 0.24, 95% CI 0.16 to 0.37, *P* < 0.001).

**Figure 4:**
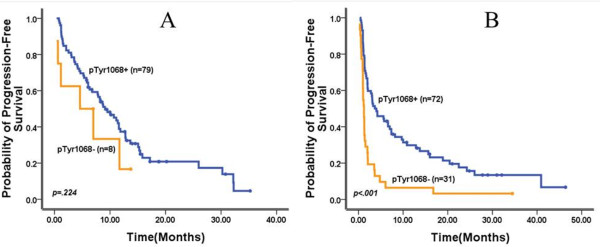
Progression-free survival curves in subgroup patients with epidermal growth factor receptor mutation positive (A) and negative (B) according to phosphorylated tyrosine (pTyr)1068 espression.

The potential role of pTyr1068 expression in predicting clinical outcomes of EGFR-TKIs therapy in patients without EGFR mutation was investigated. The results were encouraging because of the conspicuous positive correlation with a better outcome from EGFR-TKIs therapy among patients with wild-type EGFR. In subgroup of patients with wild-type EGFR, 69 patients with pTyr1068 expression (57.1.0%, 69/119) presented a prolonged PFS (4.2 months vs. 1.2 months *P* < 0.001) and improved ORR [23.2% (16/69) vs. 3.2% (1/31) *P* = 0.010) as well as DCR [69.6% (48/69) vs. 35.5% (11/31),*P* = 0.001), compared with patients with pTyr1068 negative patients (Figure 4, Table 2). Interestingly, median PFS in sixteen patients with both wild-type EGFR and pTyr1068 who have responded to EGFR-TKIs was 15.6 months (95%CI: 7.28-23.9).

#### pTyr1173 expression

Of 165 patients assessable for pTyr1173, 95 patients (57.6%) had positive pTyr1173. No significant association was observed between pTyr1173 expression and clinicopathologic characteristics including sex, age, and histology, smoking status and disease stage. Interestingly, there seemed to be a contra-correlation between pTyr1173 expression and clinical outcomes. Although differences in ORR between two groups according to pTyr1173 expression were unremarkable [27.8% (25/90) for positive VS. 37.9% (25/66) for negative, *P* = 0.123]. DCR was 64.4% (58/90) for positive vs. 88.3% (58/66) for negative (*P* = 0.007) (Table 1). And PFS was 4.8 months vs. 7.7 months, (*P* = 0.016) for negative and positive pTyr1173 which is statistically significant.

#### Interactions of biomarkers and combinational analysis

Relationship of these biomarkers and their clinical significance were analyzed. A trivial correlation between expression of pTyr1068 and EGFR mutations was observed (kappa = 0.191, *p* < 0.001). Correlations between expressions of pTyr1173, pTyr1068 and EGFR mutations (Table 3) were not significant. Analysis for combinational models of these three biomarkers suggested that in the subset of patients with an EGFR mutations, patients with both pTyr1068 positive and pTyr1173 negative expressions had superior response to TKIs as well as significantly longer PFS (*P* < 0.001), ORR (*P* < 0.001) and DCR (*P* < 0.001) (Table 4). However, no significant differences of response to gefitinib or erlotinib was observed between patients with phosphorylated Tyr1068 and Tyr1173 of EGFR (*P* > 0.05).

**Table 3:** Association between EGFR mutation and EGFR phosphorylations

**Variables (no.of patients, %)**		**EGFR mutation**			**pTyr1068**			**pTyr1173**		
		**+**	**-**	***p***	**+**	**-**	***p***	**+**	**-**	***p***
Total		92(44.9)	113(55.1)		164(80.0)	41(20.0)		95(57.6)	70(42.4)	
EGFR mutation	+				84(91.3)	8(8.7)	<0.001	41(54.7)	34(45.3)	0.297
	-				80(70.8)	33(29.2)		54(60.0)	36(40.0)	
pTyr1068	+	84(51.2)	80(48.8)	<0.001				82(61.2)	52(38.8)	0.069
	-	8(19.5)	33(80.5)					13(41.9)	18(58.1)	
pTyr1173	+	41(43.2)	54(56.8)	0.297	82(86.3)	13(13.7)	0.069			
	-	34(48.6)	36(51.4)		52(74.3)	18(25.7)				

Abbreviations: EGFR, epidermal growth factor receptor; pTyr, phophorylated tyrosine.

**Table 4:** Analysis for combinational biomarkers

**Variables**		**Combinations**								
		**Mutation+**	**Mutation-**	**Mutation+**	**Mutation-**	**Mutation+**	**Mutation-**	**Mutation+**	**Mutation-**	***P***
		**1068+**	**1068+**	**1068-**	**1068-**	**1068+**	**1068+**	**1068-**	**1068-**	-
		**1173+**	**1173+**	**1173+**	**1173+**	**1173-**	**1173-**	**1173-**	**1173-**	-
Total		35(22.4)	42(26.9)	5(3.2)	8(5.1)	30(19.2)	19(12.2)	2(1.3)	15(9.6)	-
ORR	CR + PR	16(45.7)	7(16.7)	1(20.0)	1(12.5)	20(66.7)	5(26.3)	0	0	<0.001
DCR	CR + PR + SD	28(80.0)	26(61.9)	3(60.0)	1(12.5)	29(96.7)	17(89.5)	1(50.0)	8(53.3)	<0.001
	PD	7(20.0)	16(38.1)	2(40.0)	7(87.5)	1(3.3)	2(10.5)	1(50.0)	7(46.7)	
PFS	Median (months)	6.3	3.1	4.6	1	12.8	6.6	0.4	1.4	<0.001

Abbreviations: pTyr, phophorylated tyrosine; CR, complete remission; PR, partial response; SD, stable disease; PD, progressive disease; ORR, objective response rate; DCR, disease control rate; PFS, progression-free survival, 1068 pTyr1068, 1173 pTyr1173.

Cox regression analysis was performed to determine the significance of the patients’ clinicopathologic parameters (including gender, age, smoking status, staging and pathology) and the biomarkers (EGFR mutation, expression of pTyr1173 and pTyr1068) in predicting response and progression-free survival. Only EGFR mutation and phosphorylatedTyr1068 expression were independent prognostic indicators for response and PFS. Patients harboring EGFR mutation or phosphorylatedTyr1068 expression had a better response (OR 0.244, 95%CI 0.104-0.574, *P* = 0.001; OR0.158, 95%CI 0.034-0.574, *P* = 0.020, respectively) and prolonged PFS (HR 0.422, 95% CI 0.287-0.621, *P* = 0.000 for patients with EGFR mutation; HR 0.677, 95% CI 0.502-0.969, *P* = 0.031 for the patients with phosphorylated Tyr1068).

## Discussion

Phosphorylated EGFR is an active form of EGFR protein; therefore, measurements of phosphorylated EGFR may provide useful information to determine patient’s eligibility to receive EGFR TKIs therapy [34]. This study indicated pTyr1068 or pTyr1173 might be promising predictors for patients who could benefit from EGFR-TKIs therapy. Moreover, strong evidence was provided that a phosphorylated Tyr1068 of EGFR may be an available predictive biomarker for screening population for TKIs treatment among wild-type EGFR NSCLC patients.

Hosokawa et al. reported that phosphorylated EGFR in 97 surgically resected NSCLC patients was closely correlated with EGFR protein expression, instead of EGFR mutation [35]. Okabe et al. examined the phosphorylation of Tyr845, Tyr1068, Tyr1173 and downstream molecules in vitro and showed that only Tyr1068 was constitutively phosphorylated in cell lines harboring EGFR deletion-type mutation [36]. Endoh et al. found phosphorylated EGFR status was not associated with a particular mutation type, although significant correlation of pTyr845 or pTyr1068 with EGFR mutation was observed [37]. In our study, pTyr1068 expression had a weak correlation with EGFR mutation and patients with pTyr1068 expression possessed a better response and prolonged PFS to EGFR-TKIs therapy, whereas pTyr1173 positive tumor was not correlated with EGFR mutation and had poor outcome to EGFR-TKIs therapy. Previous studies and our results indicated that there might be apparent differences between EGFR phosphorylation pattern and function of different tyrosine phosphorylation sites.

EGFR phosphorylation is likely to be of biological relevance in NSCLC [5,38]. Expression of pTyr1068 in tumor samples evaluated by IHC here exhibits a strong predictive value for EGFR-TKIs therapy, especially in patients without EGFR mutations. In the entire patient population, those with pTyr1068 expression have a significantly improved response rate and prolonged PFS compared with expression negative ones. Moreover, its predictive role is not just for efficacy in patients with concomitant EGFR mutation. Patients with pTyr1068 expression achieved a superior benefit of PFS (median 4.2 months v 1.2 months; *P* < 0.001). Especially, sixteen patients with both wild-type EGFR and pTyr1068 who have responded to EGFR-TKIs possessed a median PFS of 15.6 months (95%CI: 7.28-23.9). The results suggested pTyr1068 expression may be a supplementary predictor for EGFR-TKIs in selecting proper patients to EGFR-TKIs among those with wild-type EGFR.

Prior studies have demonstrated that the specific phosphorylation sites inside the intracellular tail often serve as docking sites for a range of proteins and initiate cascades of separate and functional distinct downstream signaling pathways [14,39], pTyr1068 is involved in MAPK and Akt pathways activation [17,20,40] being considered a marker of EGFR activation. Helfrich et al. showed not only EGFR mutant cell line (H3255) but also EGFR TKIs sensitive wild-type cell lines (H322 and Calu3) had higher pTyr1068 expression and more sensitivity to gefitinib [41]. Amann et al. showed that EGFR was constitutively phosphorylated in gefitinib-sensitive cell lines yet the level of phosphorylation of the EGFR mutant cell line was comparable with that in wild-type cells [42]. These findings suggest that EGFR activation (phosphorylation) can be triggered and then affect subsequent steps of signal transduction regardless of EGFR mutational status. In the present study, the patients with EGFR wild-type might also show high phosphorylated EGFR expression, which may account for why 10–20% of NSCLC patients in absence of EGFR mutation have responded to treatment with gefitinib or erlotinib.

Hijiya et al. investigated another autophosphorylation site Tyr1173 and found that no correlation with clinical responsiveness to gefitinib [43]. Emery et al. noted that the higher level of pTyr1173 was associated with longer time to progression (TTP) of EGFR-TKIs [29]. In contrast, there appears a negative correlation between pTyr1173 expression and clinical outcomes in our study. pTyr1173 expression is not only significantly associated with worse PFS in the univariate analysis; it also maintains independently poor prognostic significance in the multivariate analysis. Since pTyr1173 provides a docking site for Shc and is thus involved in the activation of MAPK signaling, this findings suggests that dysregulation of MAPK signaling may contribute to EGFR TKIs sensitivity in NSCLC patients. These results are also supported by the evidence from preclinical studies showing that the activation of MAPK has an antiapoptic effect on tumor cells as well as intrinsic resistance to gefitinib [30]. Further investigation will be required to address this possibility.

This study confirms the predictive value of EGFR mutation to efficacy of EGFR-TKIs in advanced NSCLC. However, according to present data, phosphorylated Tyr1068 was considered as a meaningful supplement to select NSCLC patients with wide-type EGFR who may respond to EGFR-TKIs therapy. We observed that ORR among patients without EGFR mutation was higher than expected, compared with results of previous studies [17,27,28]. One possible explanation is the racial and ethnic disparities as enrolled population mainly consisted Chinese patients, whereas most of other studies have a limited number of Chinese patients. Another possible explanation is EGFR mutation negative status in this study is determined in a diagnostic or operative procedure at time of initial presentation and may fail to fully reflect mutation status before EGFR-TKIs treatment as second- or more-line. [29].

One of the limitations of the current study is that this is a retrospective and single center study. The results need to be validated by prospective and multicenter study in the future. In addition, the half-life of phosphorylated EGFR protein is short, and therefore the specimen need to be optimally collected and processed. Otherwise phosphorylated EGFR measurements may result in misleading findings. In this study, more than 80% of samples came from our hospital and were standardized collected and stored, which could ensure the quality of specimens for phosphorylated EGFR analysis. In the future, standard platforms for collecting and detecting samples should be developed at once clinical significance of phosphorylated EGFR is validated by prospective and multicenter study.

## Conclusions

In conclusion, pTyr1068 may be a predictive biomarker for screening the population for clinical outcomes of EGFR-TKIs treatment; especially for patients with wild-type EGFR. A prospective, large-scale study is warranted.

## Abbreviations

EGFR, Epidermal growth factor receptor; EGFR-TKI, Epidermal growth factor receptor -tyrosine kinase inhibitors; MAPK, Activated protein kinase; ERK, Extracellular signal-regulated kinase; PI3K, Phosphatidylinositol 3-kinase; STAT, Signal transduction and activator of transcription; Tyr, Tyrosine; PLCγ, Phospholipase Cγ; ECOG, Eastern cooperative oncology group; ORR, Objective response rate; DCR, Disease control rate; PFS, Progression-free survival; OS, Overall survival; PD, Disease progression; CR, Complete response; PR, Partial response; SD, Stable disease; RECIST, Response Evaluation Criteria in Solid Tumors; ADC, Adenocarcinoma; HR, Hazard ratio; IHC, Immunohistochemistry; 1068, pTyr1068; 1173, pTyr1173.

## Competing interests

The authors declare that they have no competing interests.

## Authors’ contributions

All authors read and approved the final manuscript.

## Authors’ information

Supported by grants from China National Funds for Distinguished Young Scientists and the Capital Development Foundation (30772472).
